# Notes and News

**Published:** 1894-05-12

**Authors:** 


### NOTES AND NEWS.

COLLECTED AND COMMUNICATED.

MEETINGS OF THE WEEK.

Royal Caledonian Asylum, Festival Dinner, Hotel Metropole, Friday, May 18th, 6.30 p.m.

Charing Cross Hospital, Festival Dinner, Hotel Metropole, Saturday, May 19th, 7.0 p.m.

MANY facts contained in Dr. Collingridge's lecture before the Shipmasters' Society are such as would be heard with surprise by the public at large. We are accustomed to regard sailors as principally falling victims to the perils of their calling, and if in so healthy a life as the seaman's is usually regarded to be any ailment were especially common, rheumatism is the one that would most readily occur to us. Yet Dr. Collingridge told his audience that consumption claims the largest number of victims from the seafaring class. This he attributes to the small portion of air allotted to each berth in the sleeping arrangements on board ship. The maximum space provided for sailors is 72 cubic feet, whilst Dr. Collingridge points out that, from a moderate sanitary point of view, 100 cubic feet should be provided.

AN amusing if not very fruitful discussion took place at a recent meeting of the New York Academy of Medicine. The subject, opened by Dr. Achilles Rose, was as to the possibility of adopting an international language amongst medical men, which could be used at international congresses, and for all purposes of communication between medical men of different nationalities. Dr. Rose advocated the use of Greek, which, he pointed out, had the advantage of being rich in combinations, and of being a living as well as a classic language, open to adaptation to new methods and research. Dr. Leale, who expressed no faith that a universal language could be adopted for many years to come, spoke in favour of English as the dominant language. Dr. Burt agreed with Dr. Leale, whilst the President, Dr. Roosa, expressed the most practical sentiments, when he complained of the present collegiate methods of teaching any foreign language, which made it useless in application to the most willing pupil.

A BALL was given last week by the British Association of Teachers of Dancing, in aid of the Hospital Saturday Fund. As the guests numbered 250, it is hoped that the Fund will be substantially benefited.

THE Naval Hospital at Brooklyn, New York, is about to be reconstructed. Two wards with all modern improvements are to be erected to accommodate 100 patients. A new operation theatre will also form part of the scheme.

ALTHOUGH the outbreak at Lisbon has been definitely reported as cholera, the disease has shown itself in so mild a form that doubts have arisen as to its nature. Typhoid fever has also been prevalent in Lisbon, and is attributed to the contamination of the water supply. The outbreak of both kinds is rapidly declining, and there does not appear to be any present likelihood of the wide spread of Asiatic cholera from Lisbon.

AT St. Thomas's Hospital the recent additions to the Medical School are to be opened on June 9th by the President, H.R.H. the Duke of Connaught. The additions and alterations have been so arranged as to give distinction to the various sections. Thus Operative Surgery, Pathology, Biology, and Chemistry have now well-arranged separate departments. The Students' Club has been removed to the new building, whereby larger library and reading rooms are provided.

THE Ealing local authorities have, we hear, been successful in frustrating the attempt of the Metropolitan Asylums Board to erect an infectious hospital in the Perivale district. It was certainly hard measure to seize upon the prettiest bit of country, directly in the line of march of the favourite Sunday walk towards Harrow, and establish there a fever hospital which would not have been in any way for the benefit of the inhabitants of the selected district, but for those of three neighbouring parishes—Acton, Chiswick, and Hanwell. Ealing possesses an excellent little infectious hospital of its own, so the authorities may be congratulated on having made good their case against an addition, whilst we hope a more suitable site may be found elsewhere for the new hospital, which is no doubt much needed.

THE festival dinner of the Royal Eye Hospital, Southwark, will be held at the Grafton Galleries, Piccadilly, W., on Friday, May 25th, at which the Lord Mayor (accompanied by the Lady Mayoress) will preside. After the banquet the Lady Mayoress will hold a reception in the picture galleries, for which (in addition to the exhibition of pictures "Fair Women") a very attractive programme has been provided. Several prominent artistes, including Miss Ethel Winn, Miss Llelvala Davies, Miss Nettie Atkinson, Mr. and Mrs. Rutland Barrington, Mr. and Mrs. Luther Munday, Mr. Scott Fisher, and the Dilettante Vocal Quartet have kindly promised their services. It is to be regretted that at the present moment half of the beds of the hospital cannot be utilised for want of funds, and an additional yearly income of at least £2,000 is required to place the institution in full operation. Tickets for the banquet or reception can be obtained on application to the Hon. Secretary, Lieut.-Colonel Hodgson, Royal Eye Hospital, Southwark, S.E.

